# Proteomic Analysis of the Breast Cancer Brain Metastasis Microenvironment

**DOI:** 10.3390/ijms20102524

**Published:** 2019-05-22

**Authors:** Priyakshi Kalita-de Croft, Jasmin Straube, Malcolm Lim, Fares Al-Ejeh, Sunil R. Lakhani, Jodi M. Saunus

**Affiliations:** 1 Faculty of Medicine, the University of Queensland, Centre for Clinical Research, Herston 4029, QLD, Australia; p.kalita@uq.edu.au (P.K.-d.C.); m.lim@uq.edu.au (M.L.); s.lakhani@uq.edu.au (S.R.L.); 2 QIMR Berghofer Medical Research Institute, Brisbane 4006, QLD, Australia; jasmin.straube@qimrberghofer.edu.au (J.S.); fares.al-ejeh@qimrberghofer.edu.au (F.A.-E.); 3 Pathology Queensland, The Royal Brisbane & Women’s Hospital, Herston 4029, QLD, Australia

**Keywords:** brain metastasis, proteomics, tumour microenvironment, metabolism

## Abstract

Patients with brain-metastatic breast cancer face a bleak prognosis marked by morbidity and premature death. A deeper understanding of molecular interactions in the metastatic brain tumour microenvironment may inform the development of new therapeutic strategies. In this study, triple-negative MDA-MB-231 breast cancer cells or PBS (modelling traumatic brain injury) were stereotactically injected into the cerebral cortex of NOD/SCID mice to model metastatic colonization. Brain cells were isolated from five tumour-associated samples and five controls (pooled uninvolved and injured tissue) by immunoaffinity chromatography, and proteomic profiles were compared using the Sequential Window Acquisition of All Theoretical Mass Spectra (SWATH-MS) discovery platform. Ontology and cell type biomarker enrichment analysis of the 125 differentially abundant proteins (*p* < 0.05) showed the changes largely represent cellular components involved in metabolic reprogramming and cell migration (min *q* = 4.59 × 10^−5^), with high-throughput PubMed text mining indicating they have been most frequently studied in the contexts of mitochondrial dysfunction, oxidative stress and autophagy. Analysis of mouse brain cell type-specific biomarkers suggested the changes were paralleled by increased proportions of microglia, mural cells and interneurons. Finally, we orthogonally validated three of the proteins in an independent xenograft cohort, and investigated their expression in craniotomy specimens from triple-negative metastatic breast cancer patients, using a combination of standard and fluorescent multiplex immunohistochemistry. This included 3-Hydroxyisobutyryl-CoA Hydrolase (HIBCH), which is integral for gluconeogenic valine catabolism in the brain, and was strongly induced in both graft-associated brain tissue (13.5-fold by SWATH-MS; *p* = 7.2 × 10^−4^), and areas of tumour-associated, reactive gliosis in human clinical samples. HIBCH was also induced in the tumour compartment, with expression frequently localized to margins and haemorrhagic areas. These observations raise the possibility that catabolism of valine is an effective adaptation in metastatic cells able to access it, and that intermediates or products could be transferred from tumour-associated glia. Overall, our findings indicate that metabolic reprogramming dominates the proteomic landscape of graft-associated brain tissue in the intracranial MDA-MB-231 xenograft model. Brain-derived metabolic provisions could represent an exploitable dependency in breast cancer brain metastases.

## 1. Introduction

Brain relapse is a devastating complication in the course of disease for patients with breast cancer. Triple-negative breast cancers (TNBC) tend to relapse in the brain within two years of the initial breast cancer diagnosis, and patients tend to decline relatively rapidly thereafter, marked by impaired neurocognition, general poor quality-of-life and an average life expectancy of less than two years from diagnosis [1]. One reason for continued poor outcomes in this patient population is a paucity of effective systemic therapies to augment treatment with surgery and radiotherapy. More research is needed to understand the complex pathophysiology underpinning brain relapse.

Preclinical studies have mainly focused on the tumour compartment of brain metastases, for example, by applying molecular profiling technologies to matching primary and metastatic tumours from the same patients, or to ‘brain-seeking’ sub-clonal cell line derivatives generated via in vivo passage [2,3,4,5,6]. These approaches have provided important biological insights and illuminated unique selective pressures imposed by the brain microenvironment. Successful tumour clones are able to adapt by resisting oxidative stress, repurposing neurotransmitters as metabolic substrates, secreting serpins that inhibit protective brain proteases and mimicking regular neural traits [7,8,9]. Both tumour and glial compartments of the tumour microenvironment (TME) contribute to the physical and molecular frameworks required for continued outgrowth. For example, there is evidence that reactive astrocytes can transfer miRNAs targeting the PI3K pathway checkpoint *PTEN*, as well as calcium and cGAMP, leading to the release of pro-tumorigenic cytokines [10,11,12]. It has become evident that these and other brain-tumour cross-talk mechanisms help to sustain metastatic outgrowth, yet little is known about molecular alterations in the brain compartment of the TME.

With the aim of improving our understanding of metastasis-associated changes in brain tissue, we applied proteomic profiling to the mouse brain compartment of MDA-MB-231 intracranial xenografts. We also performed detailed bioinformatic analyses and investigated the expression of three of the proteins in neurosurgical samples from metastatic breast cancer patients.

## 2. Results

### 2.1. Proteomic Profiling of the Brain Microenvironment in Experimental Brain Metastases

**Figure 1:**
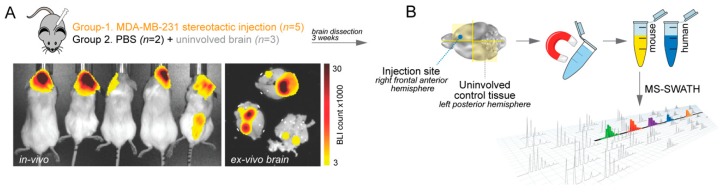
Identification of proteins with altered expression in metastasis-associated brain (MaB) tissue compared to uninvolved or injured brain tissue. (**A**) Study design and representative bioluminescence imaging (BLI) of NOD-SCID hosts and ex vivo BLI on dissected brains, three weeks after intracranial injection of MDA-MB-231 cells. (**B**) Brain tissue was dissected, purified and subjected to Sequential Window Acquisition of All Theoretical Mass Spectra (SWATH-MS).

To model brain metastasis in a controlled experimental setting in vivo, cancer cells can be introduced to the brain via different routes. We utilized direct intracranial injection to investigate the colonization stage of metastasis [13]. Single-cell suspensions of MDA-MB-231 breast cancer cells, or equal volumes of PBS (brain injury control), were delivered under anaesthesia into the right frontal anterior hemispheres of female NOD-SCID hosts (*n* = 5), assisted by a stereotactic frame and semi-automated injection apparatus. Tumour development was monitored by bioluminescence imaging (BLI) and confirmed with ex vivo brain BLI at the experimental endpoint (Figure 1A). Brains were dissected into quadrants, then mouse cells were isolated using immunoaffinity chromatography beads. We then quantified proteins in the samples using the Sequential Window Acquisition of All Theoretical Mass Spectra (SWATH-MS) discovery platform (Figure 1B).

**Figure 2:**
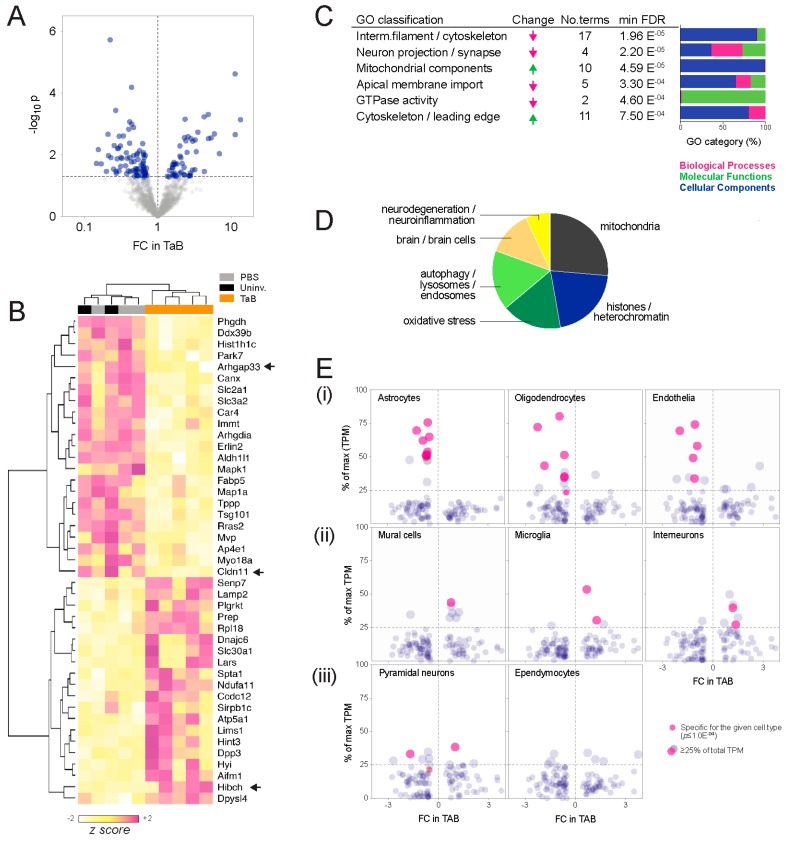
Brain proteomic landscape in the MDA-MB-231 intracranial xenograft model. (**A**) Volcano plot showing 125 differentially abundant proteins, including 50 that were significantly more abundant, and 75 less abundant in graft-associated brain tissue compared to controls (blue; *p* < 0.05). (**B**) Heatmap and cluster dendrogram showing the most statistically significant alterations (*p* < 0.01). Arrows indicate those selected for in situ validation. (**C**) Enrichment analysis summary. Six major ontology classes were over-represented by the alterations—four amongst low abundance proteins and two amongst high abundance proteins. Also shown are the number of terms collapsed into major classes, minimum false discovery rate (FDR) of terms in each, and proportions of gene ontology (GO) processes, molecular functions and cellular components driving the enrichment scores. (**D**) High-throughput text mining summary showing PubMed article topics associated with the protein list (FDR < 0.05). (**E**) Meta-analysis of associations with mouse brain cell types. Dot plots show RNA levels as a percentage of total expression for each transcript, versus the fold-change (FC) observed at the protein level in TAB tissue. For species strongly associated with particular cell types (defined as ≥25% of total expression across all types and/or a significant relationship in an ANOVA test), changes in TAB could indicate corresponding changes in abundance of that cell type. (i–iii) Cell type-specific biomarkers trending towards increases, decreases or no overall change, respectively.

SWATH-MS identified 1223 mouse proteins, of which 125 (10.2%) were detected at significantly different levels in MDA-MB-231 graft-associated brain tissue compared to controls (*p* < 0.05; Figure 2A,B; Table S1). To investigate the biological relevance of these differentially abundant brain proteins, we took two complementary approaches: Enrichment analysis, and analysis of neuronal and neuroglial biomarkers.

- Ontology enrichment analysis. Analysis of interconnectivity amongst the differential proteins using the STRING database [14] showed significant enrichment of known protein-protein interactions, implying biological relatedness (high-confidence interactions *p* = 3.4 × 10^−8^; Figure S1). To investigate whether these networks represent particular bio-ontologies, we performed functional enrichment analysis and consolidated the associated ontology terms according to semantic similarity using REVIGO [15]. This indicated that components, processes and functions typically associated with neurons decreased in graft-associated brain tissue, and those associated with metabolic reprogramming or cell migration increased (Figure 2C; Table S2). Cellular components (CC, compartments or stable macromolecular complexes) were the predominating category, and construction of a similarity network from the CC terms revealed two major clusters: One comprising mitochondria and vesicular elements, and a second containing mainly cytoskeletal and neural projection elements (Figure S2; Table S2). Consistent with this, high-throughput PubMed text mining indicated that the differentially abundant proteins have been most frequently studied in the contexts of mitochondria, histones, oxidative stress and autophagy (Figure 2D; Table S3).
- Cell composition analysis. The proteomic landscape of graft-associated brain tissue could also reflect changes in the relative abundance of different cell types, so we investigated this using meta-analysis of single-cell RNA sequencing (scRNAseq) data generated by Zeisel and colleagues from pooled cortical and hippocampal mouse brain tissue [16]. While based on transcriptomic rather than proteomic output, it is the largest (*n* = 3005 cells) and most comprehensive expression mouse brain cell dataset available. Considering the proteomic data as a survey of tissue composition, we compared the changes observed in the SWATH-MS experiment with cell type specificity at the RNA level, defined by statistical association (ANOVA test; *p* ≤ 1.0 × 10^−4^), as well as the proportion expressed by each cell type (Figure 2E). This established glial markers (e.g., Claudin-11 (oligodendrocyte-specific protein; OSP) and Aldh1l1 (10-formyltetra-hydrofolate dehydrogenase), associated with astrocytes and oligodendrocytes, respectively [17,18,19,20,21]), confirming the meta-analysis as a valid way to identify cell type-associated transcripts. Overall, transcripts associated with astrocytes, oligodendrocytes and endothelia were less abundant at the protein level in TAB, while those associated with mural cells (pericytes and vascular smooth muscle), microglia and interneurons were more abundant.

Overall, these data indicate that the proteomic landscape of MDA-MB-231 graft-associated brain tissue is dominated by features associated with metabolic and cell migratory activity, reflecting a combination of specific neuroinflammatory processes and altered cell composition. Of note, many of the proteins identified have been implicated in metabolic and oxidative stress. For example, in graft-associated brain there was a 2.4-fold increase in the autophagy marker Lamp2 (Lysosome-associated membrane protein 2; *p* = 3.5 × 10^−3^); a 1.5-fold decrease in the redox-sensitive chaperone, Park7 (Protein/nucleic acid deglycase DJ-1; *p* = 2.5 × 10^−3^), for which germline mutations are associated with early-onset Parkinson’s Disease; and a 4.9-fold increase in Lims1 (LIM and senescent cell antigen-like-containing domain protein; also known as PINCH; *p* = 4.9 × 10^−4^), which binds and is associated with accumulation of hyperphosphorylated tau proteins.

### 2.2. Independent Validation of HIBCH, Cldn11 and Arhgap33

Next, we selected three differentially abundant proteins for validation using fluorescent multiplex immunohistochemistry (fmIHC) analysis of an independent xenograft cohort (*n* = 5). Even with fluorescent detection, IHC has a narrower dynamic range than SWATH-MS, so we chose proteins with the most significantly altered expression for which antibodies were commercially available: (1) Arhgap33 (Rho GTPase Activating Protein 33), a sorting nexin (*SNX26*) implicated in dendritic spine formation and intracellular trafficking of TrkB [22,23]; (2) Cldn11 (claudin-11), produced by oligodendrocytes and a key component of myelin-stabilizing tight junctions in the CNS [24]; and (3) HIBCH (3-Hydroxyisobutyryl-CoA Hydrolase), an enzyme involved in degradation of branched chain amino acids (BCAA) like valine.

**Figure 3:**
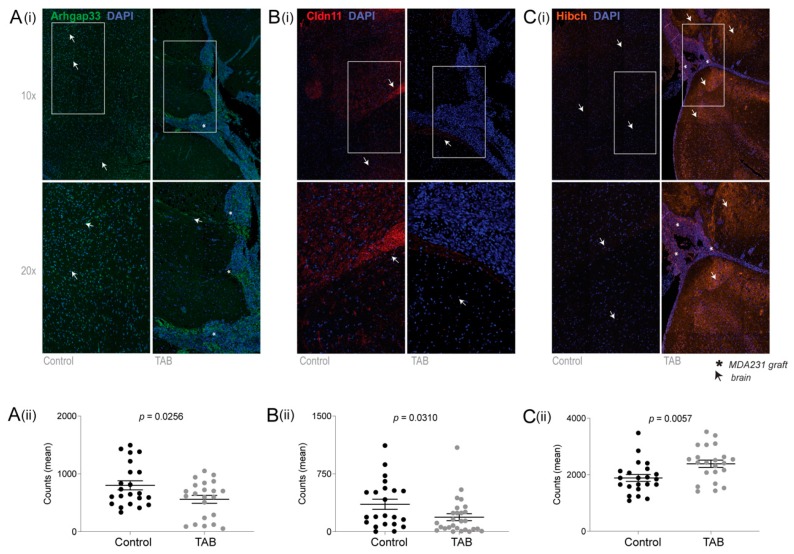
Representative fluorescent multiplex immunohistochemistry (fmIHC) analysis of HIBCH, Arhgap33 and Cldn11 expression in wounded (control) and tumour-associated brain (TaB) tissues. (i) Fluorescent images for each marker (**A**–**C**) counterstained with DAPI are shown at two different magnifications. (ii) Mean fluorescence counts (± standard error) within 22 regions-of-interest (ROIs; 9 mm^2^) across whole-brain coronal section images for each mouse (*n* = 5/cohort). Unpaired Mann–Whitney tests were used to compare controls and TAB for each marker; *p* values indicated.

Formalin-fixed, paraffin-embedded (FFPE) coronal mouse brain sections were analysed by fmIHC (Figure 3), including co-staining with an antibody against the reactive astrocyte marker, glial fibrillary acidic protein (GFAP), to aid segmentation of tumour and brain compartments in the digital images prior to fluorescence analysis (Figure S4). All three antibodies react against human and mouse proteins, and accordingly we detected HIBCH and Arhgap33 in both brain and xenograft compartments. In brain, staining for Arhgap33 and Cldn11 was comparatively lower in TAB compared to the controls, confirmed by quantifying the fluorescent signal across multiple regions-of-interest (*p* = 0.026/0.031, respectively; Figure 3A,B). Conversely, HIBCH was considerably higher in TAB (Figure 3C; *p* = 0.0057).

### 2.3. Expression of HIBCH, CLDN11 and ARHGAP33 Proteins in Human Brain Metastases

**Figure 4:**
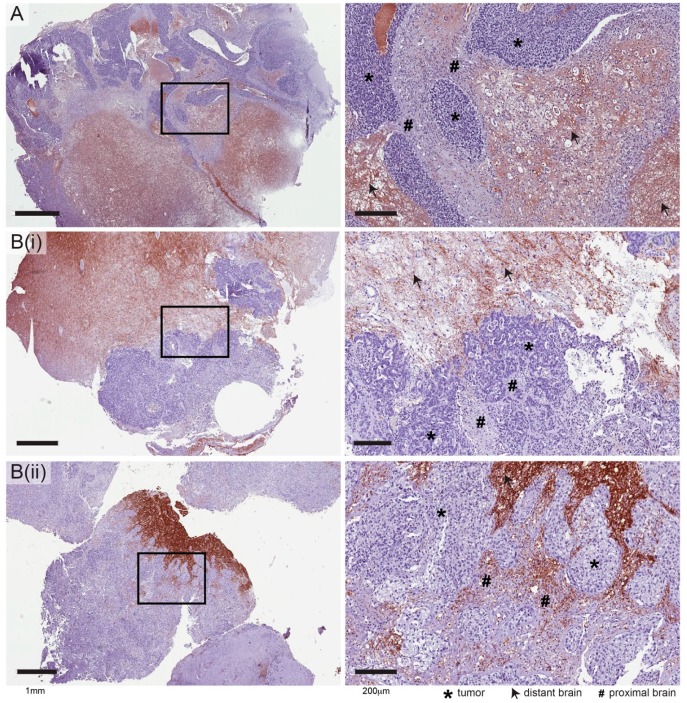
Representative IHC staining of human triple-negative breast cancer brain metastases with antibodies against ARHGAP33 (**A**) and CLND11 (**B**). Two examples are shown for Claudin-11: (i) Broad tumour margins pushing into white matter, where neurites (arrows) are positive, while tumour-associated gliosis is negative (#); and (ii) tumour architecture resulting in a more infiltrative growth pattern, where claudin-11-positive neurites can be seen proximal to tumour nests (#). Left, low magnification; right, enlargement of the boxed regions.

To investigate the potential relevance of these candidates in humans, we analysed their expression in craniotomy specimens from metastatic TNBC patients. Surgical resection is not routine in breast cancer clinical management, but is considered for some patients when debulking could provide symptom relief [25]. Also, since neurosurgery is conservative, only a proportion of these rare samples is suitable for TME studies requiring an uninvolved brain tissue component. After reviewing H&E-stained sections of TNBC brain metastases from the Brisbane Breast Bank [26], we identified five cases suitable for analysis of both proximal (tumour-associated) and distant (uninvolved) brain tissue regions. IHC analysis was performed on serial sections using the same antibodies as xenograft experiments. For Arhgap33, staining was associated with neurites, which were generally displaced by tumour-associated gliosis (reactive change of glial cells in response to damage to brain tissue), resulting in overall lower levels in regions proximal to tumour nests (e.g., Figure 4A). Similarly, Claudin-11 staining was generally excluded from TAB (Figure 4B(i)), though it was occasionally positive in regions with an atypical infiltrative growth pattern (Figure 4B(ii)).

**Figure 5:**
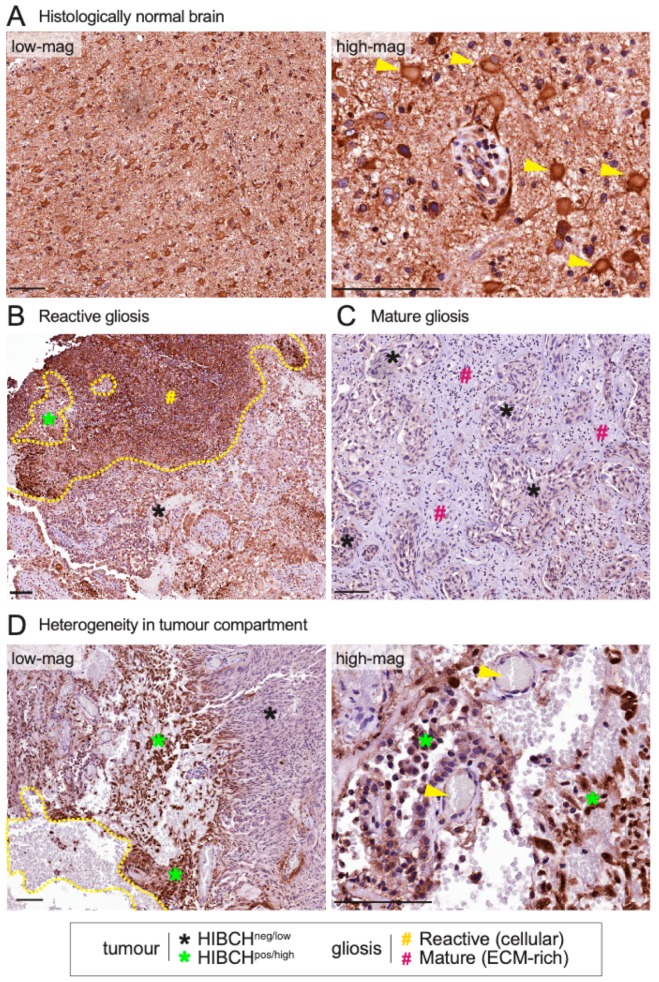
IHC analysis of HIBCH expression in human triple-negative breast cancer brain metastases —key observations from five cases. (**A**) Representative staining in histologically normal brain tissue, at low and high magnification, showing large neurons with strong cytoplasmic expression (yellow arrows) (**B**) Representative strong HIBCH staining in reactive gliosis. The yellow line demarcates gliotic from metastatic tissue. (**C**) Extracellular matrix (ECM)-rich gliosis surrounding HIBCH-negative tumour cells. (**D**) Heterogeneity in the tumour compartment. Yellow line encapsulates haemorrhagic tissue and the enlargement shows small cerebral blood vessels (yellow arrows). Scale bars: 100 μm.

Expression of HIBCH was observed in both brain and tumour compartments, with variable staining intensity in each. Consistent with RNA data (Figure S3), HIBCH was ubiquitously expressed in histologically normal brain tissue, with uniform staining throughout the neuropil and strong cytoplasmic expression in large neurons (Figure 5A). Conversely, expression in the metastatic brain TME was heterogeneous. In general, areas of reactive gliosis associated with metastatic cells were marked by very strong HIBCH staining (Figure 5B), while lower levels were detected in mature, extracellular matrix (ECM)-rich gliotic tissue (Figure 5C). The tumour compartment was also heterogeneous, and interestingly, positivity was often localized to tumour margins, vascularized and haemorrhagic areas that might be expected to provide metabolic substrates (Figure 5D).

## 3. Discussion

Molecular cross-talk between tumour and other cells in the surrounding microenvironment is a key factor driving metastatic outgrowth [7,8,10,11,27,28,29]. To our knowledge, this is the first study to directly investigate the impact of tumour development on the proteome of neighbouring brain cells in vivo. Using the SWATH-MS discovery platform, we identified 125 proteins that were significantly altered in tumour-associated compared to uninvolved and traumatically injured brain tissue. This includes the astrocytic basement membrane protein laminin α2 (Lama2), which is implicated in blood-brain-barrier integrity, and others found was less abundant in the metastatic brain TME of MDA-MB-231.BR6, JIMT-1.BR3 and SUM190.BR3 intracardiac xenograft models [30].

We considered the MS data at cell and tissue levels, analysing ontology enrichment and cell type biomarkers. Overall, the data indicate that the brain proteomic landscape of intracranial MDA-MB-231 xenografts is characterized by a complex neuroinflammatory reaction, involving considerable metabolic reprogramming, oxidative stress and activation of autophagy. Deregulation of cellular bioenergetics is a hallmark of cancer, but in contrast to tumour cell intrinsic adaptations, our understanding of parallel “metabolic evolution” in the parenchymal compartment is fairly limited. 

The application of in situ validation in this study provided important perspectives around the MS findings. Of the proteins validated orthogonally by fmIHC, in situ analysis suggested that Arhgap33 and Claudin-11 were probably less abundant in TAB due to displacement of neural tissue by the expanding tumour. Conversely, increased levels of HIBCH seemed to be in keeping with the metabolically active state of gliotic foci. Regions of TAB tissue with low HIBCH expression were also observed. However, these were distinguished by higher ECM-glia ratios and were organized around tightly packed tumour cell nests, possibly reflecting a more advanced state of tissue remodelling where the energetic needs of the glial compartment are lower [31].

HIBCH is a mitochondrial inner membrane protein with specific hydrolase activity for (S)-3-hydroxyisobutyryl-CoA, an intermediate in the valine degradation pathway. Valine is an essential, branched-chain amino acid (BCAA) catabolized in the brain as a parallel source of energy to glycolysis [32]. Steady-state HIBCH levels are normally fairly high due to rapid turnover [33], and unlike other amino acids, valine can be catabolized without suppressing autophagy [34,35]. Induction of HIBCH in response to increased metabolic demands may therefore explain why it was the most strongly induced protein in the SWATH experiment, and the supra-normal staining intensities we observed in gliotic foci of craniotomy samples. 

Chen and colleagues investigated proteomic features of brain-tropic MDA-MB-231 cells, and reported that most of the differentially expressed proteins function in metabolism [36]. In particular, there was evidence that the pentose phosphate pathway is important for meeting energy and purine biosynthesis requirements in brain metastases, utilizing glutamine and BCAA substrates in brain interstitial fluid [36]. Therefore, a possible consequence of HIBCH induction in reactive glia could be that valine degradation intermediates and/or products are transferred to the tumour compartment. The evolutionary purpose of such a response in the CNS might be related to tissue remodelling, but in the metastatic TME, could provide fuel and promote continued tumour outgrowth. There is certainly a strong precedent for this from studies implicating vesicular transport of specific growth factor and miRNA cargoes from tumour-associated glia [10,37].

Key strengths of this exploratory study are its agnostic and precise approach to candidate identification, the use of traumatically injured brain tissue as a control rather than uninvolved brain alone, and in situ comparison to human craniotomy specimens. However, the study was not without limitations, which were mainly related to its scale, with statistical power restricted by relatively small numbers of discovery and validation samples. The proteins identified here therefore likely represent the most obvious changes in a larger network. Finally, a key point of difference made clear by the human sample work is that xenografts are short-term models of colonization lacking mature, ECM-rich gliotic scars that form after significant tissue remodelling. 

Links between some of the candidates and ontologies identified here, with current knowledge about brain metastasis pathogenesis are reasonably logical, for example, metabolic reprogramming in reactive glia, and a potential role for valine catabolism in both neuroinflammatory and metastatic tissue. Nevertheless, the biological significance and potential clinical relevance of many remains to be established. Functional studies are needed to identify aspects of the neuroinflammatory response that directly promote outgrowth [38], such as the provision of metabolic substrates and growth factors via secretion, extracellular vesicles or direct transfer. While immunoaffinity separation of single cell suspensions was applied successfully here, microdissection of TAB tissue is an alternative approach that would also capture extracellular proteins.

## 4. Materials and Methods

### 4.1. Experimental Brain Metastases for Tumour Microenvironment Profiling

The human triple-negative breast cancer cell line, MDA-MB-231, was purchased from ATCC, verified by STR profiling and confirmed to be free of mycoplasma. Cells were maintained in vitro in DMEM supplemented with 10% (vol/vol) foetal calf serum and antibiotic/antimycotic (ThermoFisher Scientific, Waltham, MA, USA), trypsinized, counted (Countess^®^, ThermoFisher Scientific) and prepared as single-cell suspensions on ice. Five-week-old female NOD-SCID mice were used for the study (Animal Resources Centre, Western Australia). Under isoflurane anaesthesia, mice were immobilized in a stereotactic frame and the skull over the right hemisphere was exposed via skin incision. Using a high-speed air-turbine drill (World Precision Instruments, Sarasota, FL, USA) with defined coordinates, a small hole was slowly drilled in the skull. A total of 2 × 10^5^ cells was injected into the right cerebral hemisphere to a depth of 3 mm (total volume 2.5 μL). The skull was then sealed using histocompatible bonewax and vetbond tissue adhesive (Provet, Brisbane, QLD, Australia). Over the next 3 weeks, tumour development was monitored by bioluminescence imaging (BLI) using the IVIS100 live animal imaging system (Caliper Life Sciences, Waltham, MA, USA), 1 minute after intraperitoneal injection of VivoGlow Luciferin (50 mg/kg; Promega, Madison, WI, USA). Mice were humanely sacrificed, and brains removed for analysis after 3 weeks. All animal procedures were performed in accordance with our approval from the QIMR Berghofer animal ethics committee.

### 4.2. Cell Separation

Mouse brains were dissected into quadrants (Figure 1A), then mouse cells were isolated from tumour-associated, mock and normal brain tissue homogenates on ice using affinity-based magnetic bead separation (Adult brain dissociation kit, Miltenyi Biotec, Bergisch Gladbach, Germany) according to the manufacturer’s instructions, and isolates were immediately snap-frozen in liquid nitrogen. Further sample preparation, Mass Spectrometry (MS)-SWATH analysis and data pre-processing were undertaken at the Australian Proteome Analysis Facility (APAF), according to established protocols [39] (see below).

### 4.3. Sample Preparation and Mass Spectrometry (MS)

Cells in 9 mL of liquid medium were concentrated to 700 μL using buffer exchange in 3 kDa spin columns (Vivaspin turbo 15, Sartorius, Göttingen, Germany), which were initially conditioned with 100 mM TEAB buffer containing 1% sodium deoxycholate. The protein concentration in the sample was determined using the Direct Detect assay (Millipore, Burlington, MA, USA), then 50 μg (at 1 μg/L) was reduced in 10 mM dithiothreitol for 1 hour at 56 °C, followed by alkylation in 25 mM iodoacetamide at room temperature for 30 min. Samples were digested with 1 μg of trypsin for 16 h at 37 °C, then sodium deoxycholate was precipitated from the samples with 1% formic acid. After centrifuging for 10 min at 10,000× *g*, peptide-containing supernatants were dried by speed-vac, further purified in 2% acetonitrile and 0.1% formic acid, then subjected to LC-MS/MS characterization.

For information-dependent acquisition (IDA), peptide samples (3 μg) were subjected to nanoLC MS/MS analysis. Pre-concentration and de-salting were done on a reverse-phase trap at 5 μL/min for 5 min, then peptides were eluted on linear solvent gradients of 5.5–33% of solvent B (90% acetonitrile, 9.9% water and 0.1% formic acid) over 120 min at 600 nL/min. Eluents were subjected to positive ion nano-flow electrospray analysis in IDA mode. A time-of-flight (TOF) survey scan was acquired (*m/z* 350–1500, 0.25 s), with the 10 most intense, multiply charged ions (2+–4+; >150 counts/s) sequentially subjected to MS/MS (spectra accumulated for 100 ms in the range *m/z* 100–1800, with rolling collision energy). LC-MS/MS data were mapped to the Uniprot *Mus musculus* database (54,451 mouse proteins, 18-Sept-2017 [40]) with ProteinPilot (v5.0; Sciex) using ParagonTM in thorough mode. Carbamido-methylation of cysteine residues was selected as a fixed modification. An Unused Score cut-off was set to 1.3 (95% confidence for identification). The extracted SWATH peak areas were normalized to the total peak area for each run after subtracting albumin peptides contributed by contaminating blood/plasma, thus expression values for each species represent a proportion of all non-albumin proteins detected.

### 4.4. Identification of Differentially Abundant Proteins

Expression values were log2 transformed to guarantee quasi-normal distribution, then assessed for reproducibility by looking at the intensity distribution and correlation between samples using Pearson correlations. Differential expression analysis between five tumour-associated and five control samples (three uninvolved brain samples and two “injured” samples) was performed using the Limma package [41]. Unsupervised hierarchical clustering with a complete linkage algorithm was performed on differentially abundant proteins, specifically, the 1-Pearson correlation dissimilarity metric of transformed data (Bayesian t-test *p* < 0.01).

### 4.5. Bioinformatics

Analysis of protein-protein interactions and reference publication enrichment were performed on 125 differentially abundant proteins (*p* ≤ 0.05), using the STRING database [14]. For the top 100 publications where subsets of the differentially abundant proteins are mentioned more frequently than expected by chance (*p* ≤ 0.05; Table S3), common themes were extracted according to the number of times individual nouns appeared in the titles. Gene ontology (GO) enrichment analyses were performed on separate lists of higher (*n* = 50) and lower (=75) abundance proteins using STRING. Significantly enriched GO IDs (cellular components, biological processes and molecular functions with *p* ≤ 0.01) were classified by semantic similarity using the REVIGO tool [15].

For analysis of mouse brain cell type biomarkers, we mined a single-cell RNAseq dataset derived from 3005 mouse brain cells [16]. To compare the relative abundance of different transcripts between cell types, data were transformed to account for differences in the total pool of mRNA/cell, converting absolute RNA counts to counts/million transcripts (TPM). Expression of transcripts encoding differentially abundant proteins was averaged across cells within the nine major categories identified by Zeisel et al. [16] (hippocampal and cortical pyramidal neuron categories were pooled as there were no significant differences for any of the transcripts of interest). Transcripts were grouped according to the cell type exhibiting maximal expression (Figure S3), and the most cell type-specific transcripts were also identified using 2-way ANOVA tests with Tukey’s correction for multiple comparison testing (Prism v8). Median expression trends of maximal expression-categorized transcripts (Figure 2E(i)) were consistent with the trends of the most cell type-specific transcripts (Figure 2E(ii)).

### 4.6. Fluorescent Multiplex Immunohistochemistry (fmIHC) and Multispectral Imaging

Four-micron thick coronal brain tissue sections (*n* = 5 for each group) were stained with cocktails of primary antibodies (Table S4) and detected using tyramide signal amplification technology (Opal kit, Perkin Elmer, Waltham, MA, USA) according to the manufacturer’s instructions. The reactive astrocyte marker, glial fibrillary acidic protein (GFAP), was included as a positive control for delineating tumour-associated brain tissue (Figure S4). Slides were scanned using the Vectra 3.0 Automated Quantitative Pathology Imaging System and analysed using inForm software (v2.4.1; Perkin Elmer). Tissues were segmented into tumour and brain regions, then five 3 mm × 3 mm regions of interest (ROI) were drawn in each compartment for fluorescence acquisition. Mean ROI pixel counts from TAB and control samples were compared using Mann–Whitney unpaired t-tests (Prism, v8).

### 4.7. Immunohistochemistry (IHC) Analysis of Craniotomy Specimens

We performed a small survey of candidate expression in human breast cancer brain metastases in collaboration with the Brisbane Breast Bank [26], according to the conditions of our human research ethics approval from committees of the Royal Brisbane and Women’s Hospital and The University of Queensland. Five cases were selected based on the triple-negative status of the primary tumour, and suitability for TME analysis based on histopathological review of hematoxylin and eosin-stained sections. A list of antibodies and IHC conditions used is in the Supplementary Table S4. IHC was performed on 4 μm formalin-fixed, paraffin-embedded whole sections using the Mach1 Universal HRP-Polymer Detection Kit (BioCare Medical, Pacheco, CA, USA) according to the manufacturer’s instructions. Briefly, sections were deparaffinized with xylene and hydrated in a series of graded ethanol (95%–70%) to water. Heat-induced antigen retrieval was performed using a decloaking chamber™ (BioCare Medical) with sodium citrate buffer (0.01 M, pH 6.0) for 30 min at 95 °C. Sections were then treated with 0.3% hydrogen peroxide for 10 min to block endogenous peroxidases. Non-specific antibody staining was blocked with MACH1 Sniper blocking reagent (BioCare Medical). Primary antibody diluted in TBS was applied on slide for O/N at 4 °C in a humidified slide chamber. For rabbit primary antibodies, MACH1 anti-rabbit secondary antibody conjugated to horseradish peroxidase was applied for 30 min at room temperature. Diaminobenzidine (DAB) chromogen substrate was applied for 1–5 min. Lastly, slides were counterstained with hematoxylin for 30 s and cover-slipped with DPX mountant (Sigma-Aldrich, St Louis, MO, USA). For analysis, slides were scanned at 40x magnification using the Aperio AT Turbo (Leica Biosystems, Wetzlar, Germany) and digital images were reviewed by three observers (PKC, JMS, and ML).

## Supplementary Materials

Supplementary materials can be found at https://www.mdpi.com/1422-0067/20/10/2524/s1.

## Abbreviations

FFPE	Formalin fixed paraffin embedded
HIBCH	3-hydroxyisobutyryl-CoA hydrolase
Cldn11	Claudin 11
TSA	Tyramide signal amplification
GFAP	Glial fibrillary acidic protein
BSA	Bovine serum albumin
RT	Room temperature
