# Supplementary material for: Proteomic Analysis of the Breast Cancer Brain Metastasis Microenvironment
Source: Int J Mol Sci. 2019 May 22;20(10):2524. doi: 10.3390/ijms20102524 (PMC6567270; doi:10.3390/ijms20102524)
Supplement: Supplementary file 1 [file ijms-20-02524-s001.zip › ijms-484609 suppl figures.pdf]

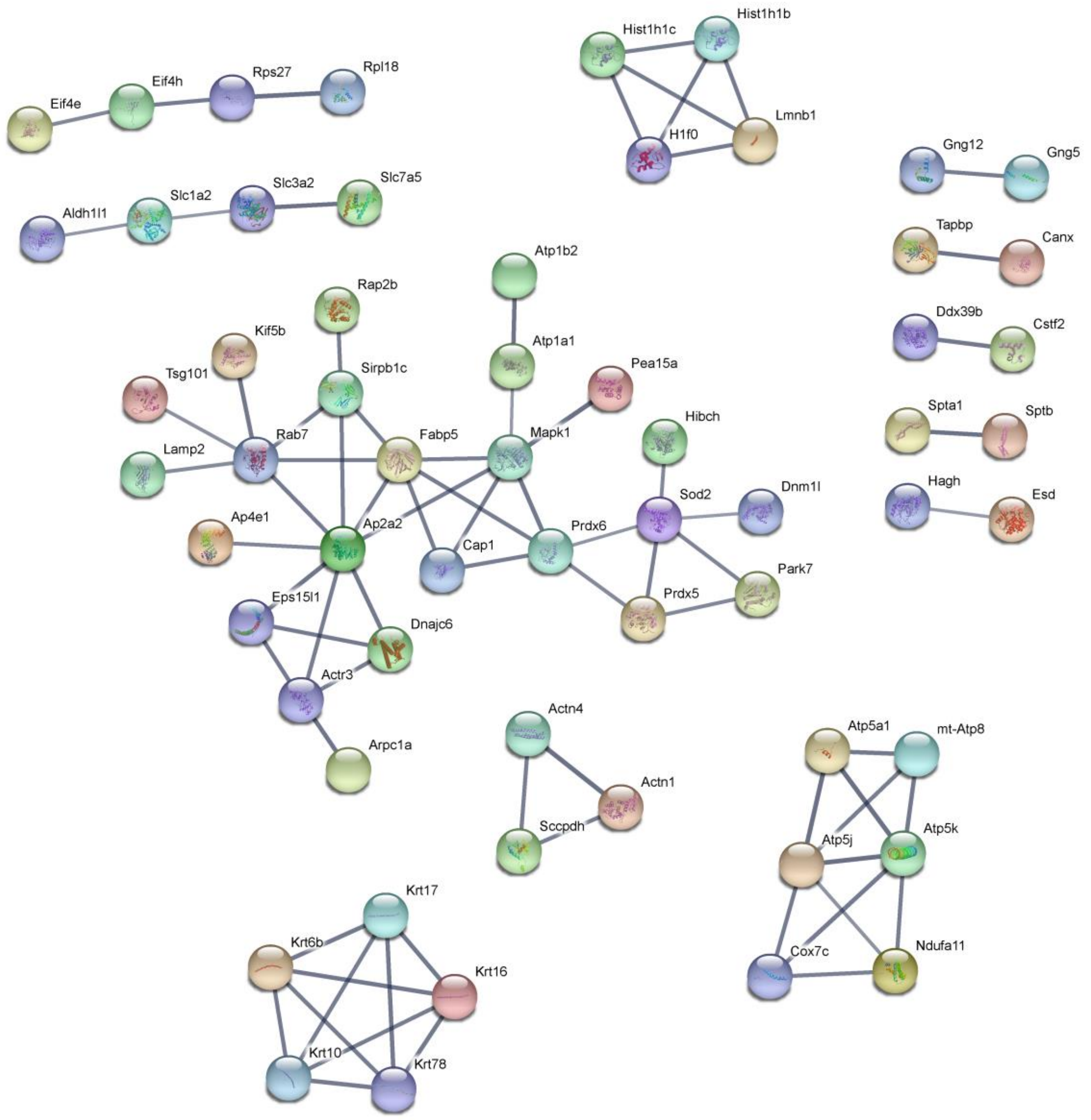

Figure S1. High confidence protein-protein interactions identified using the STRING database. Based on an interaction score of ≥0.7 (maximum 1.0), 60/125 (48%) of the differentially abundant protein have previously documented evidence of a physical interaction, implying biological relatedness. Interaction scores range from 0 to 1.0, and are calculated from genome neighborhood & co-occurrence, gene fusions, co-expression, experimental/biochemical data, and association in curated databases including PubMed. The number of connections (edges) identified was 76, which is significantly higher than the number expected by chance (38; $p = 3.44E^{-08}$).

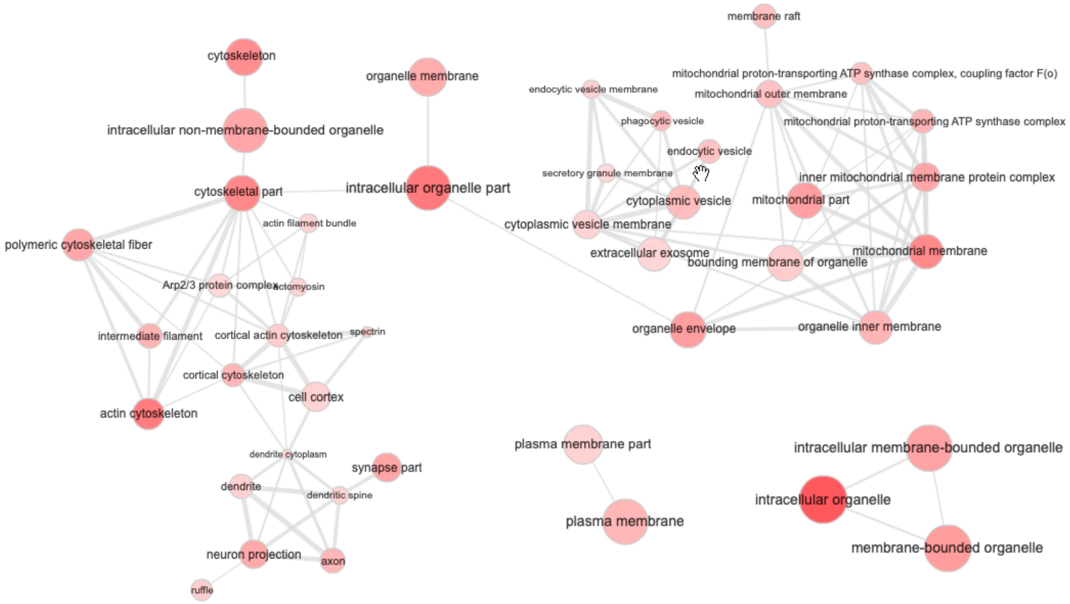

Figure 2. Cytoscape cellular component network constructed from 125 differentially abundant proteins identified in tumor-associated brain tissue. Two major component clusters make up the bulk of the network – cytoskeletal components of neural projections (left) and mitochondrial and vesicular components (right). Connections and distance are proportional to semantic similarity of GO titles and descriptions. Node size is inversely proportional to the false discovery rate.

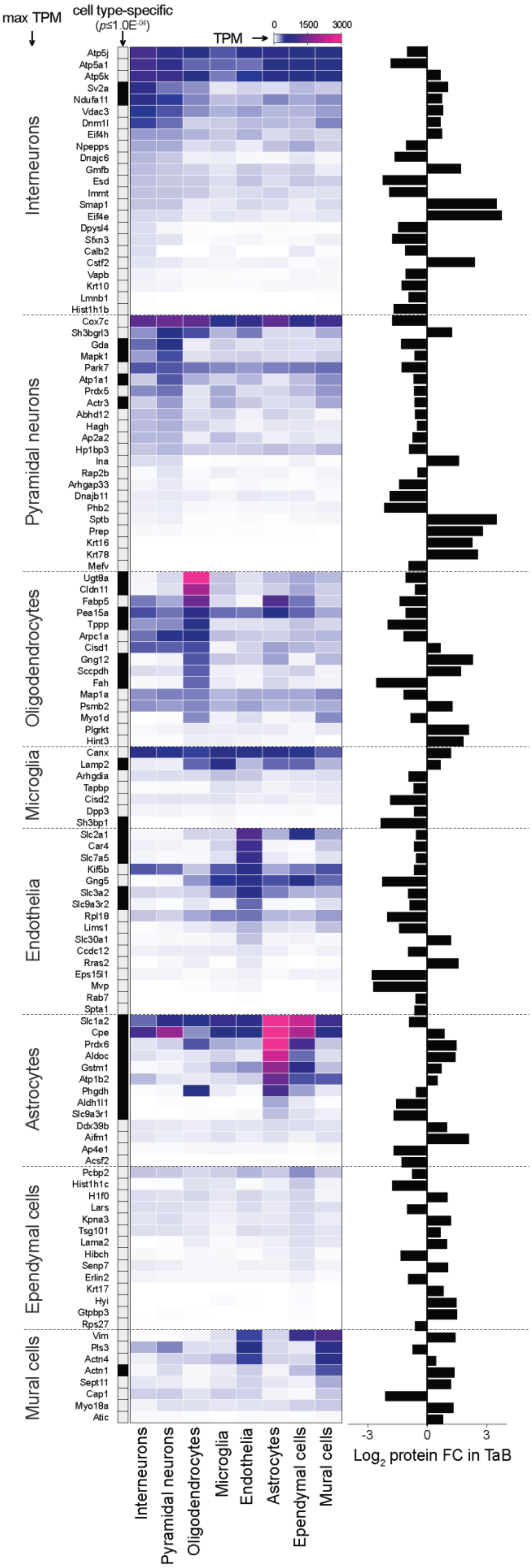

max TPM
cell type-specific
(p≤1.0E-04)
TPM
0
1500
3000
Interneurons
Atp5j
Atp5a1
Atp5k
Sv2a
Ndufa11
Vdac3
Dnm1l
Eif4h
Npepps
Dnajc6
Gmfb
Esd
Immt
Smap1
Eif4e
Dpysl4
Sfxn3
Calb2
Cstf2
Vapb
Krt10
Lmnb1
Hist1h1b
Pyramidal neurons
Cox7c
Sh3bgrl3
Gda
Mapk1
Park7
Atp1a1
Prdx5
Actr3
Abhd12
Hagh
Ap2a2
Hp1bp3
Ina
Rap2b
Arhgap33
Dnajb11
Phb2
Sptb
Prep
Krt16
Krt78
Mefv
Oligodendrocytes
Ugt8a
Cldn11
Fabp5
Pea15a
Tppp
Arpc1a
Cisd1
Gng12
Sccpdh
Fah
Map1a
Psmb2
Myo1d
Plgrkt
Hint3
Microglia
Canx
Lamp2
Arhgdia
Tapbp
Cisd2
Dpp3
Sh3bp1
Endothelia
Slc2a1
Car4
Slc7a5
Kif5b
Gng5
Slc3a2
Slc9a3r2
Rpl18
Lims1
Slc30a1
Ccdc12
Rras2
Eps15l1
Mvp
Rab7
Spta1
Astrocytes
Slc1a2
Cpe
Prdx6
Aldoc
Gstm1
Atp1b2
Phgdh
Aldh1l1
Slc9a3r1
Ddx39b
Aifm1
Ap4e1
Acsf2
Ependymal cells
Pcbp2
Hist1h1c
H1f0
Lars
Kpna3
Tsg101
Lama2
Hibch
Senp7
Erlin2
Krt17
Hyi
Gtpbp3
Rps27
Mural cells
Vim
Pls3
Actn4
Actn1
Sept11
Cap1
Myo18a
Atic
Interneurons
Pyramidal neurons
Oligodendrocytes
Microglia
Endothelia
Astrocytes
Ependymal cells
Mural cells
-3
0
3
Log2 protein FC in TaB

Figure S3. Heatmap showing average expression of differentially abundant proteins at the RNA level in cell types of the mouse brain (meta-analysis of [15]). Transcripts are grouped according to the cell type exhibiting maximal expression, and ranked in descending order for each category. TPM = transcripts per million. Cell type specificity was determined using an ANOVA test; transcripts significantly associated with one particular cell type are indicated with black tiles. Bar chart shows protein fold change (FC) in tumor-associated brain (TaB) tissue.

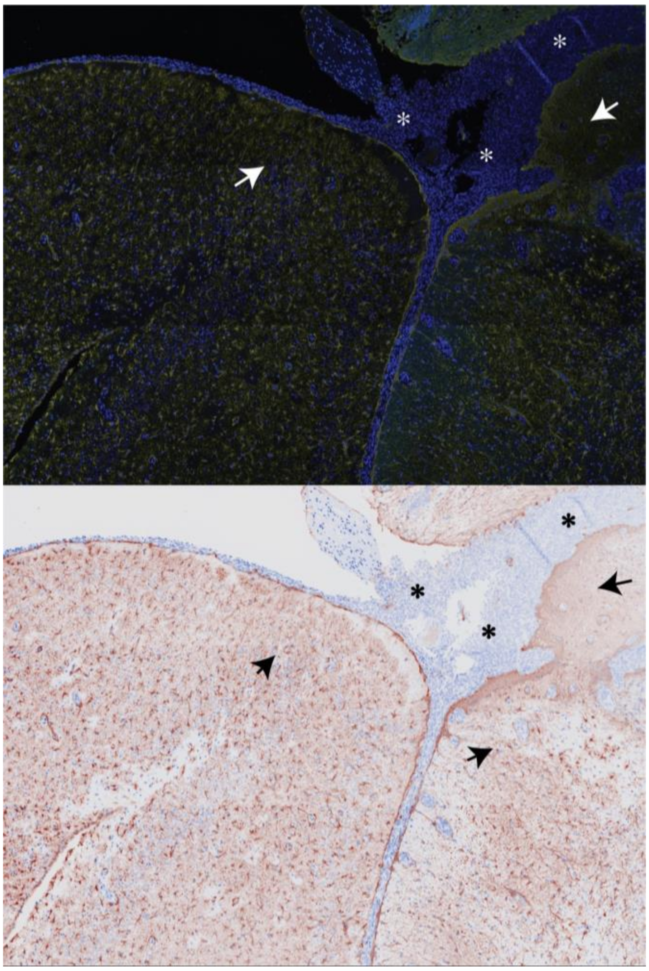

Figure 4. Example of GFAP expression in tumor-associated brain tissue. GFAP (yellow; upper panel) was multiplexed with investigational antibodies to guide tissue segmentation in xenograft images prior to fluorescence analysis. Lower panel: simulated diaminobenzidine tetrahydrochloride (DAB) detection of the same section, incorporating bright-field exposure so that morphological features can also be considered during tissue segmentation. *Arrows: brain tissue; asterisks, tumor*.
